# GLP-1 and GLP-2 as intestinal reparative therapies in inflammatory bowel disease: mechanisms, translation, and clinical opportunity

**DOI:** 10.3389/fgstr.2026.1790420

**Published:** 2026-05-18

**Authors:** Joseph J. Lee, Pratikiran Bajgain, Amy L. Lightner

**Affiliations:** 1 Department of Colorectal Surgery, Scripps Clinic, La Jolla, CA, United States; 2 Naval Medical Center San Diego, Department of Surgery, San Diego, CA, United States; 3 Department of Molecular & Cellular Biology, The Scripps Research Institute, La Jolla, CA, United States; 4 Department of Immunology and Microbiology, The Scripps Research Institute, La Jolla, CA, United States; 5 Calibr-Skaggs, The Scripps Research Institute, La Jolla, CA, United States

**Keywords:** Crohn’s disease, GLP, GLP-1, GLP-2 receptor agonist, IBD - inflammatory bowel disease, ulcerative colitis

## Abstract

Inflammatory bowel disease remains a chronic condition in which a substantial proportion of patients fail to achieve durable clinical remission despite advances in therapy. Although the treatment landscape has expanded to include biologics and small-molecule agents, contemporary management is increasingly focused on altering the course of the disease rather than relying on symptom control alone. Central to this shift is the recognition that intestinal reparative therapy, defined by objective endpoints such as endoscopic and histologic healing, is strongly associated with improved long-term outcomes. Emerging mechanistic data highlight epithelial repair pathways as actionable therapeutic targets. Among these, glucagon-like peptides secreted by intestinal enteroendocrine cells have gained attention for their roles in epithelial integrity and inflammation modulation. This review synthesizes basic science and translational evidence supporting glucagon-like peptide-1, glucagon-like peptide-2, and dipeptidyl peptidase-IV inhibition as emerging therapeutic concepts in inflammatory bowel disease. Furthermore, it provides a critical appraisal of recent human observational data, distinguishing metabolic benefits from intrinsic disease-modifying effects, and outlines the practical clinical friction points and prospective trials necessary to validate these pathways in future treatment paradigms.

## Background

Inflammatory bowel disease (IBD) is an idiopathic condition characterized by chronic relapsing inflammation in the gastrointestinal tract. IBD is associated with the dysregulation of the symbiotic relationship between the commensal ecosystem and the mucosal immune system (1–3). Patients with IBD exhibit recurrent remission and relapse with symptoms that significantly impact their quality of life (4, 5). The compounding prevalence of IBD projects an exponential increase in the number of patients in the coming decades (6).

Crohn’s disease (CD) and ulcerative colitis (UC) are the two major phenotypes of IBD, and they differ meaningfully in their biology, barrier injury patterns, and epithelial restitution mechanisms. CD is characterized by transmural inflammation that can affect the entire gastrointestinal tract, often presenting with specific phenotypes such as stricturing, inflammatory, or perianal disease. Conversely, UC is limited to the mucosal layer of the colon and rectum (7, 8). Despite these phenotypic differences, both conditions involve a loss of gut epithelial barrier integrity (1). A healthy intestinal barrier, composed of a protective mucus layer and a physical intestinal epithelium, is essential for maintaining mucosal immune homeostasis (9–11). At the level of the intestinal epithelium, a healthy barrier possesses a self-repair mechanism to preserve barrier function (12). Disruption to this barrier triggers a compensatory immune response and induces inflammation (13, 14).

A considerable number of patients with IBD fail to achieve durable clinical remission. The treatment landscape has evolved significantly from conventional aminosalicylates, corticosteroids, and immunomodulators. Approved advanced therapies now include anti-tumor necrosis factor alpha (anti-TNF-α) agents, anti-integrins, cytokine inhibitors targeting IL-12/23 and IL-23, Janus kinase (JAK) inhibitors, and sphingosine-1-phosphate receptor (S1PR) modulators. Crucially, IBD management has shifted from merely controlling symptoms to halting disease progression through “intestinal reparative therapy.” In this context, true repair is strictly defined by objective endpoints such as endoscopic healing and histologic healing, rather than subjective clinical remission. The normalization of intestinal permeability, while not formally included among STRIDE-II targets, represents a desirable future endpoint that may further refine the assessment of barrier restoration. Achieving these stringent endpoints, as outlined in the Selecting Therapeutic Targets in Inflammatory Bowel Disease (STRIDE-II) consensus (15), demonstrates disease modification and is associated with reduced relapse rates, lower corticosteroid dependence, and increased surgery-free survival (16–18). Given its critical role, current mechanistic studies are increasingly focused on devising potential treatments that target wound healing and restore barrier integrity (19).

Research into mucosal healing has highlighted pathways involving interleukin-20 (IL-20), claudin-2 (CLDN2), and notoginsenoside R1 (NGR1) (13, 20–23). Among emerging targets, glucagon-like peptides (GLPs), which are bioactive compounds secreted by intestinal L-cells, present a distinct mechanism focused on barrier integrity and wound healing that is fundamentally separate from standard systemic anti-inflammatory strategies.

**Figure 1:**
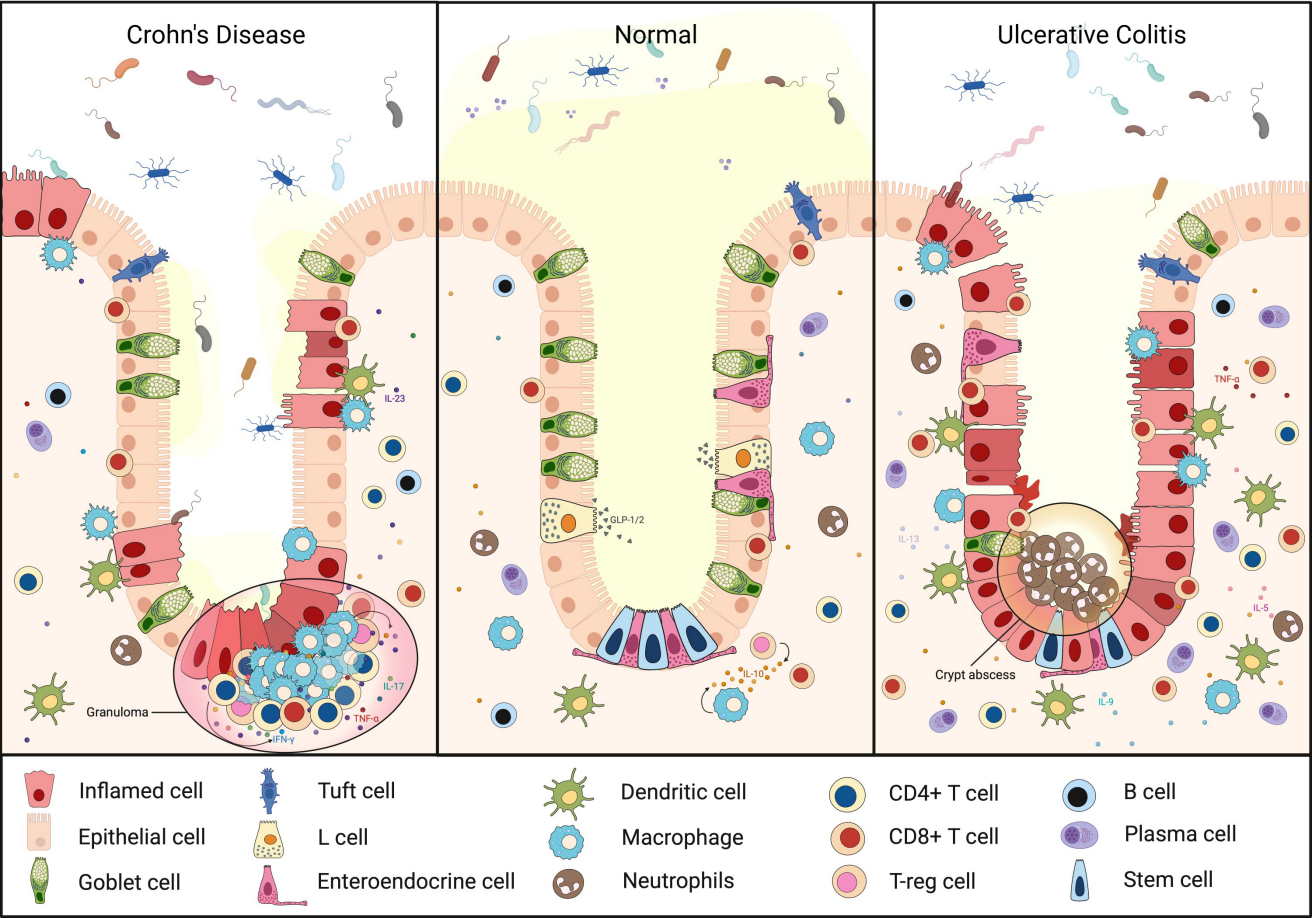
Immune cell distribution in the intestinal crypts under homeostasis and inflammatory bowel disease. Under homeostasis (middle), there is an organized distribution of stem cells, goblet cells, tuft cells, L-cells, and lymphocytes with an intact epithelial barrier. In CD (left), there is transmural, patchy inflammation with granuloma clusters in the lamina propria. In UC (right), there are broad patches of mucosal inflammation with the lumen filled with neutrophils forming crypt abscesses (24).

**Figure 1** illustrates the differences in immune cell distribution within the intestinal crypts under homeostatic conditions compared with the inflammatory microenvironments of CD and UC.

## What are GLPs?

**Figure 2:**
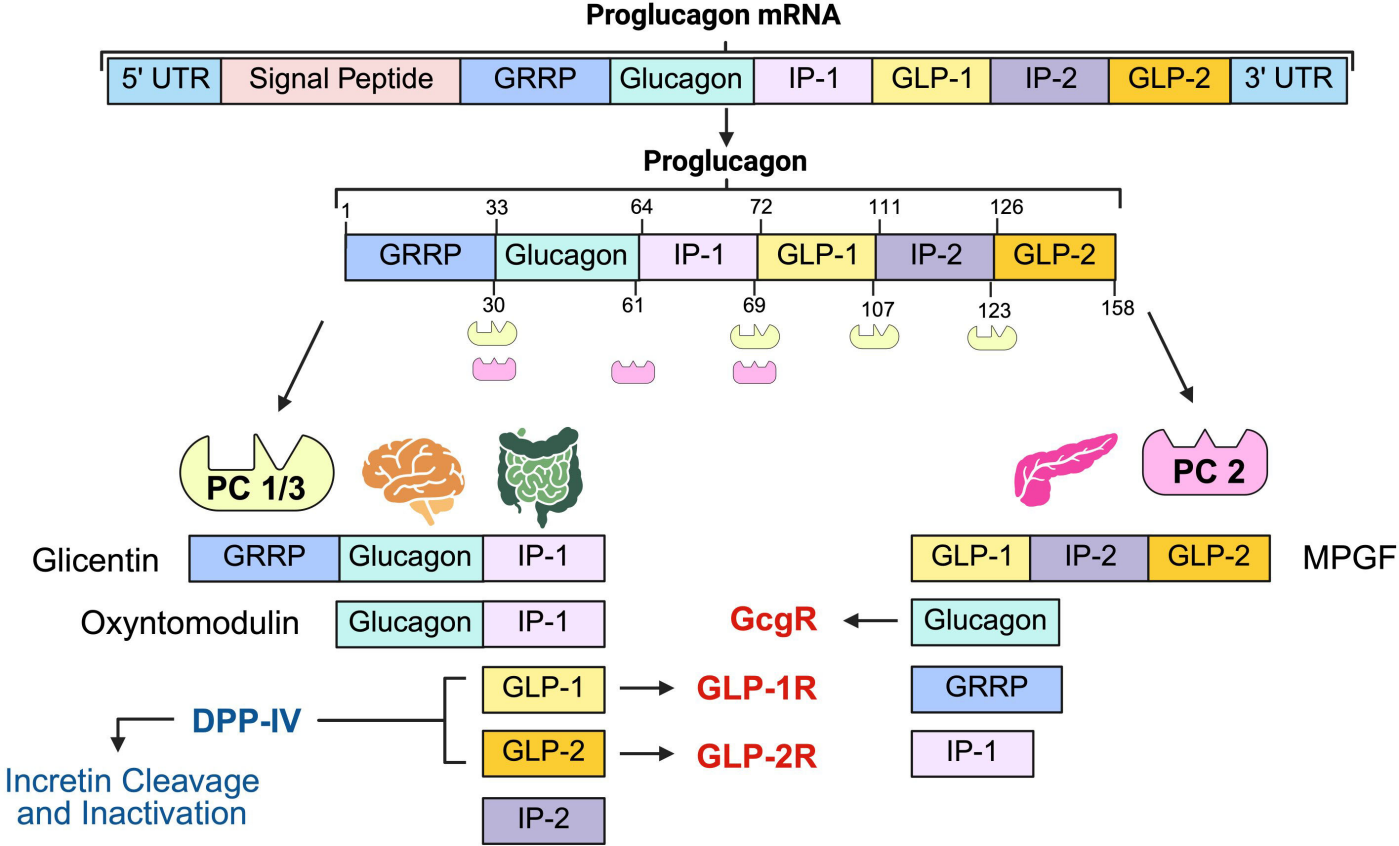
Post-translational processing of proglucagon mRNA with PCs. In the brain and gut, PC1/3 cleaves proglucagon to form glicentin, oxyntomodulin, GLP-1, GLP-2, and IP-2 peptides. In the pancreas, PC2 cleaves proglucagon forming glucagon, GRRP, and IP-1 (31). Abbreviations: PC, prohormone convertase; GLP, glucagon-like peptide; IP, intervening peptide; GRRP, glicentin-related pancreatic polypeptide; GLP-1R, glucagon-like peptide-1 receptor; GLP-2R, glucagon-like peptide-2 receptor; DPP-IV, dipeptidyl peptidase IV; GcgR, glucagon receptor, UTR, untranslated region.

The proglucagon gene (gcg) encodes for glucagon as well as two glucagon-like peptides, GLP-1 and GLP-2 (25, 26). These peptides are part of the proglucagon-derived peptide (PGDP) family, formed through the differential post-translational processing of proglucagon by tissue-specific prohormone convertases (PC). PC2 in pancreatic alpha-cells cleaves proglucagon to generate glucagon, while PC1/3 in gut enteroendocrine L-cells yields GLP-1 and GLP-2 alongside other fragments (14, 27–30). **Figure 2** summarizes the tissue-specific post-translational processing of proglucagon by PC1/3 and PC2, the resulting peptide products, and their receptor targets.

### GLP-1

GLP-1 is a highly regulated, post-translational product of proglucagon in the gut, secreted primarily in response to nutrient uptake, as its plasma concentrations are very low in the fasting state (32–35). GLP-1 acts through its receptor, GLP-1R, which is a class B G-protein-coupled receptor (GPCR). Ligand binding on the extracellular domain triggers an intracellular cascade that increases cyclic AMP (cAMP) production via adenylate cyclase (36–42). GLP-1R is widely distributed across the pancreatic islets, brain, heart, and gastrointestinal tract (43–46).

Beyond its well-established role in normalizing plasma glucose in type 2 diabetes (47, 48), GLP-1 exerts mucosal protective effects, including promoting mucosal healing, pathogen defense, and facilitating weight loss (49–53). In murine models, GLP-1 levels rose rapidly in response to gut barrier injury, suggesting a role in acute intestinal restitution (54). GLP-1 signaling also directly downregulates proinflammatory cytokines in mucosal immune cells (55), consistent with a targeted hierarchy of mechanisms that prioritizes epithelial defense and local immune modulation over systemic immunosuppression.

### GLP-2

GLP-2 is a 33-amino acid peptide secreted from intestinal L-cells in a 1:1 ratio with GLP-1. It has a short circulating half-life of approximately seven minutes due to rapid degradation by dipeptidyl peptidase IV (DPP-IV) (14, 56–59). GLP-2 acts through GLP-2R, a highly specific GPCR located in the gastrointestinal tract, pancreas, and central nervous system (60–63).

Importantly, GLP-2R is not expressed on enterocytes. Instead, it is localized to intestinal subepithelial myofibroblasts and enteroendocrine cells. This indicates that its proliferative and cytoprotective effects on the gut epithelium are mediated indirectly via paracrine signaling (64–67). The best characterized of these paracrine intermediates are keratinocyte growth factor (KGF), which mediates GLP-2-induced colonic growth, and insulin-like growth factor-1 (IGF-1), which is essential for GLP-2’s trophic effects in the small intestine (68). Subepithelial myofibroblasts expressing GLP-2R increase IGF-1 mRNA expression and secretion in response to GLP-2 stimulation, and genetic deletion of the intestinal epithelial IGF-1 receptor abolishes GLP-2-induced crypt cell proliferation in mice (69, 70). Additional downstream mediators include ErbB ligands such as epiregulin and vasoactive intestinal polypeptide (VIP) (68). While the IGF-1 and KGF pathways have been reproducibly demonstrated across multiple murine models, direct human validation of these intermediates in IBD tissue remains limited, representing a key translational gap.

GLP-2 is recognized as a key intestinal growth factor capable of enhancing mucosal proliferation, improving nutrient absorption, and maintaining gut barrier integrity (71). Exogenous GLP-2 administration increases gut weight and is highly effective in the clinical management of short bowel syndrome (14, 72, 73). However, chronic stimulation of these paracrine myofibroblast pathways in IBD raises theoretical safety concerns regarding stricturing, fibrosis risk, and the necessity for rigorous dysplasia surveillance, which remain critical hurdles for long-term clinical development. This concern is particularly relevant for patients with stricturing CD, in whom up to 50% develop fibrostenotic complications within 10 years of diagnosis (74). Because GLP-2 acts through subepithelial myofibroblasts, which are the same cell population implicated in intestinal fibrogenesis, the risk-benefit profile of GLP-2 agonism may differ substantially between mucosal UC, inflammatory ileal CD, and stricturing or penetrating CD phenotypes, and this phenotypic stratification should be a required feature of future trial design.

## Dipeptidyl peptidase IV

DPP-IV, also known as CD26, is a serine protease that degrades both GLP-1 and GLP-2. This cleavage rapidly inactivates the incretins, limiting their effectiveness in promoting intestinal homeostasis (75–78). Inhibition of DPP-IV elevates endogenous GLP concentrations, but DPP-IV also independently modulates inflammatory mediators, including stromal cell-derived factor 1 (SDF-1) and regulated upon activation, normal T cell expressed and presumably secreted (RANTES) (79–81). DPP-IV activity is upregulated during inflammatory responses, and preclinical models of colitis have demonstrated that DPP-IV inhibitors reduced the expression of proinflammatory cytokines and improved histological outcomes (77).

## Emerging hypothesis and therapeutic rationale

Translational studies have sought to map the protective properties of GLP-1 and GLP-2 to clinical applications for patients with IBD. To contextualize the preclinical findings described below, it is important to note that different GLP-mediated mechanisms map to distinct clinical biomarkers and endpoints. Epithelial restitution and barrier integrity changes would be expected to manifest as improvements in intestinal permeability assays and endoscopic healing scores, such as Simple Endoscopic Score for Crohn’s Disease [SES-CD] or Mayo Endoscopic Score (15, 19). In contrast, anti-inflammatory effects on immune cell populations would be reflected by reductions in fecal calprotectin and serum C-reactive protein (CRP) (15, 82). Separating these readouts in future trials will be essential to determine whether GLP agonism produces true reparative effects versus indirect metabolic benefits. Additionally, the translational relevance of these findings likely differs by disease phenotype; mucosal barrier repair programs may be most directly applicable in UC and inflammatory luminal CD, while the trophic and proliferative effects of GLP-2 require more cautious evaluation in stricturing or penetrating CD given the fibrosis considerations discussed above.

Animal models have mapped specific target cell types to downstream restitution programs. In a 2017 study, a nanomedicine formulation of GLP-1 was used to treat dextran sulfate sodium (DSS)-induced colitis in mice. The treatment preserved colon length and reduced histological epithelial injury, outcomes linked to the suppression of IL-1β expression (83). Another study found that GLP-1R activation in Brunner’s glands upregulated barrier defense genes, an effect entirely absent in GLP-1R knockout mice (53).

GLP-1 also modulates local inflammation through specialized cell populations. Intraepithelial lymphocytes (IELs), a distinct lineage of T cells residing within the epithelial layer, express high levels of GLP-1 receptors. Yusta and colleagues demonstrated that GLP-1 analogs suppress proinflammatory cytokine expression in IELs through cAMP-mediated signaling (84). Furthermore, GLP-1 analogs exert immunomodulatory effects on colonic smooth muscle cells, which are important for maintaining gut motility and modulating the local production of proinflammatory cytokines within the intestinal wall. In response to lipopolysaccharide stimulation, exendin-4 reduced the expression of IL-1β and TNF-α in these cells (85). It is important to note that the upregulation of IL-33 observed in some GLP-1 studies does not universally guarantee mucosal defense, as IL-33 can also drive potent Th2 inflammatory responses depending on the tissue context (53). Models of visceral hypersensitivity further highlight how GLP-1 analogs improve mucosal barrier integrity (52).

**Figure 3:**
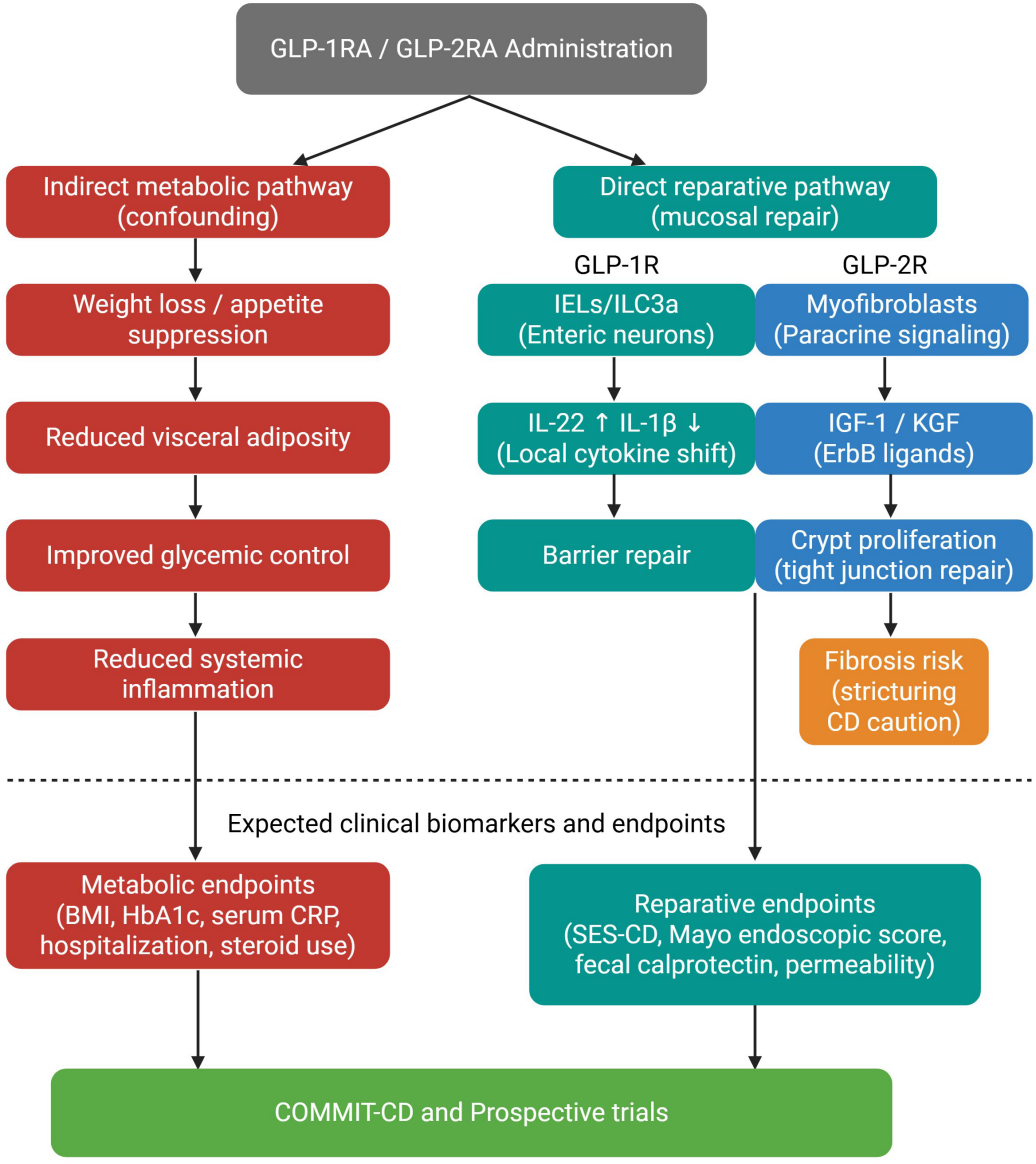
Proposed mechanistic framework distinguishing indirect metabolic pathways from direct epithelial reparative programs of GLP-1 and GLP-2 in inflammatory bowel disease. The left arm illustrates the indirect metabolic pathway, in which GLP-1RA-mediated weight loss, reduced visceral adiposity, and improved glycemic control lead to decreased systemic inflammation. These effects are unavoidably confounded with direct drug action in current observational studies. The right arm depicts the direct reparative pathway, in which GLP-1R activation on intraepithelial lymphocytes and group 3 innate lymphoid cells drives local cytokine modulation and barrier repair, while GLP-2R activation on subepithelial myofibroblasts stimulates IGF-1 and KGF paracrine signaling to promote crypt cell proliferation and tight junction restoration. The amber box indicates the theoretical fibrosis risk associated with GLP-2 agonism in stricturing CD. Below the dashed line, expected clinical biomarkers and endpoints are mapped to each pathway, with prospective trials such as COMMIT-CD required to distinguish between the two arms (92).

In 2024, Sun and colleagues investigated GLP-1 receptor agonists in a DSS-colitis model. Liraglutide treatment enhanced the activity of group 3 innate lymphoid cells (ILC3s), leading to elevated levels of interleukin-22 (IL-22), a key cytokine essential for epithelial regeneration (86). Regarding GLP-2, Gu and colleagues evaluated a modified GLP-2 analog in mice, demonstrating that it reduced disease severity by restoring epithelial junctions and barrier integrity (72). These mucosal regenerative effects have been consistently demonstrated in experimental animal models of IBD (87), complementing their currently approved use of GLP-2 analogs in short bowel syndrome (88, 89). A combined approach utilizing dual agonists or co-loaded nano-capsules has completely restored mucosal integrity in L-cell ablated mice, underscoring the complementary reparative roles of GLP-1 and GLP-2 (90, 91). **Figure 3** provides an integrative schematic distinguishing the indirect systemic metabolic benefits of GLP-1RAs from their direct epithelial reparative programs, highlighting where confounding by weight loss is unavoidable versus where true mucosal repair mechanisms are expected to dominate.

## Clinical translation and human data taxonomy

The translation of these mechanisms into human clinical data requires a strict evidence taxonomy to avoid conflating metabolic improvements with intrinsic disease modification. Current human data can be categorized into three distinct areas: safety and tolerability, metabolic outcomes, and signals for disease modification. **Table 1** organizes recent studies investigating the use of GLP analogs and DPP-IV inhibitors in IBD by study design, treatment arm, key results, and evidence classification with risk-of-bias assessment.

**Table 1:** Recent studies investigating GLP analogs and DPP-iv inhibitors use in inflammatory bowel disease.

Type	Study design	Treatment arm	Key results	Evidence class and risk of bias	Reference
Clinical	Multicenter retrospective cohort comparing adults with IBD and obesity treated with GLP-1RAs vs. untreated controls. (n, 320)	Weekly injection of GLP-1 RAs (semaglutide, liraglutide, tirzepatide)	Effective weight loss. Decreased risk of any-cause hospitalization. No patients in the GLP-1 group required surgery, unlike the control group.	Level III (Retrospective). High risk of unmeasured confounding and lack of endoscopic endpoints.	(Desai et al., 2024) (98)
Clinical	Retrospective cohort of IBD patients treated with GLP-1RAs analyzing adverse events and biomarker response. (n, 120)	Injection of GLP-1 RAs (semaglutide and liraglutide)	C-reactive protein levels decreased after 1 year (P, 0.005). No differences in endoscopic scores. Gastrointestinal side effects common (11.5%).	Level III (Retrospective). High risk of confounding by weight loss.	(Anderson et al., 2022) (94)
Clinical	Double-blind crossover study of UC patients with IPAA and medically refractory high bowel frequency. (n, 8)	Daily GLP-1RA (liraglutide) injections vs. placebo	Liraglutide reduced daily bowel frequency by >35% compared to placebo. Calprotectin stable. Nausea reported.	Level II (Small crossover). Moderate risk of bias due to small sample size.	(Herfarth et al., 2024) (118)
Clinical	Case report of a UC patient with IPAA pouchitis. (n, 1)	Dual GLP-1/GIP (tirzepatide)	Weight loss; altered thiopurine metabolism requiring 6-mercaptopurine dose reduction.	Level IV (Case report). High risk of bias.	(Klein et al., 2024) (107)
Epidemiology	Population-based study analyzing DPP-IV inhibitor risk of IBD.	DPP-IV inhibitors	No increased risk of IBD compared to other antidiabetic agents.	Level III (Observational). Moderate risk of bias.	(Wang et al., 2019) (119)
Murine	Indomethacin colitis (rat model)	GLP-2 analog (glepaglutide)	Reduced inflammation, increased intestinal mass.	Preclinical. N/A	(Skarbaliene et al., 2023) (87)
Murine	DSS-induced colitis (mouse model)	GLP-1 RA (liraglutide)	Dose-dependent improvement in colitis.	Preclinical. N/A	(Saadoun et al., 2025) (120)
Murine	DSS-induced colitis (mouse model)	Modified GLP-2 dimer	Superior reduction in colitis severity, improved histology.	Preclinical. N/A	(Gu et al., 2018) (72)
Murine	DSS-induced colitis (mouse model)	DPP-IV inhibitors (sitagliptin)	Attenuated colitis, improved colon length via potentiation of GLP-2 action.	Preclinical. N/A	(Ning et al., 2020) (121)

IBD, Inflammatory Bowel Disease; CD, Crohn’s Disease; UC, Ulcerative Colitis; GLP-1RA, Glucagon-like peptide-1 receptor agonist; IPAA, Ileal pouch-anal anastomosis; GI, Gastrointestinal; DSS, Dextran sulfate sodium.

### Safety and tolerability

The strongest evidence indicates that GLP-1RAs exhibit reassuring safety in patients with IBD. Retrospective cohort studies show that these agents do not increase the risk of IBD flares or gastrointestinal-specific adverse events compared to the general population (93–96). Notably, studies have indicated a significant reduction in C-reactive protein levels one year after the initiation of GLP-1RA therapy (82). Across the available retrospective cohorts, treatment discontinuation rates due to adverse events were generally low. Anderson and colleagues reported that approximately 11.5% of patients experienced gastrointestinal side effects, though specific discontinuation rates attributable to these events were not consistently reported (94). Clarke and colleagues similarly noted that the majority of patients tolerated GLP-1RA therapy without requiring treatment cessation due to IBD-specific complications (96). Importantly, a meta-analysis of 14 clinical trials evaluating gastrointestinal adverse effects of GLP-1RAs found that long-acting agents had lower rates of nausea and vomiting than short-acting formulations; however, this analysis was conducted in general obesity and diabetes populations, not IBD-specific cohorts, and its findings may not directly translate to patients with underlying intestinal inflammation (97).

### Metabolic outcomes

GLP-1RAs consistently yield significant weight loss and glycemic control in patients with IBD and comorbid obesity or diabetes. Agents such as semaglutide and tirzepatide improve metabolic parameters reliably without worsening baseline inflammatory disease activity (95, 96), particularly benefiting patients with coexisting metabolic conditions (98). While gastrointestinal adverse effects exist, they generally do not lead to higher hospitalization rates due to disease flares (99).

### Signals for disease modification

Evidence for true disease modification remains low-certainty and hypothesis-generating, with recent observational signals serving as a foundation for potential disease modification rather than established therapeutic benefit (100). A nationwide Israeli Epi-IIRN cohort of 3,737 patients with IBD and type-2 diabetes found that GLP-1 analogs lowered the composite risk of steroid dependence, hospitalization, and surgery. However, the protective effect was strongest in patients with obesity and absent in non-obese patients, strongly suggesting that benefits are heavily confounded by weight loss and metabolic improvement rather than direct gut anti-inflammation (101). Similarly, a Danish registry study observed lower risks of corticosteroid initiation and hospital admissions among GLP-1RA users (102).

A critical real-world evidence (RWE) appraisal of these cohorts reveals significant limitations. Inclusion criteria often blended new and prevalent drug users, introducing immortal time bias. Comparator groups varied between studies; the Danish cohort used other antidiabetic agents as the reference, while the Israeli study compared GLP-1RA users against non-users within the IBD-diabetes population, making cross-study comparison difficult. Adjustment strategies relied on measured confounders such as HbA1c and BMI but could not fully account for time-varying confounding by indication. Clinicians may have preferentially prescribed GLP-1RAs to patients with better-controlled IBD (101, 102). Furthermore, these cohorts represented populations with relatively mild disease severity proxies, indicated by low rates of prior advanced therapy use. Crucially, none of these studies utilized adjudicated endoscopic assessments, meaning true “reparative” mucosal healing could not be verified (101, 103). Consequently, while the data reflect reassuring safety, they do not conclusively prove an intrinsic therapeutic benefit for IBD. In a smaller cohort of patients with more severe CD phenotypes, including 17% with perianal involvement and 24% with penetrating or fistulizing disease, numerical reductions across outcomes were observed in the GLP-1RA group, but these differences did not reach statistical significance (104). This finding reinforces the concept that visceral and subcutaneous adipose tissues contribute differently to systemic inflammation, and that metabolic control plays a distinct role in disease pathophysiology (105, 106).

## Managing clinical friction points

If clinicians are to leverage GLP-1RAs, they must manage practical friction points. GLP-1RAs slow gastric emptying, which can alter the absorption of oral IBD medications. A recent case report documented toxic elevations in 6-thioguanine levels following tirzepatide initiation (107). Clinicians should proactively recheck thiopurine metabolites within four to six weeks of starting a GLP-1RA to prevent unexpected toxicity.

Beyond thiopurines, the delayed gastric emptying induced by GLP-1RAs may theoretically affect other oral IBD medications. A systematic review of drug-drug interactions with GLP-1RAs found that most co-administered oral drugs did not experience clinically significant changes in total exposure (AUC), although reduced peak concentrations (Cmax) and delayed time to peak (Tmax) were commonly observed, particularly for drugs with high solubility and permeability (108). For IBD-specific agents, pH-dependent formulations such as delayed-release mesalamine and enteric-coated budesonide may be of particular concern, as prolonged gastric retention could alter their site-specific release profiles. While formal pharmacokinetic studies of GLP-1RA co-administration with these IBD drugs have not been conducted, clinicians should monitor for unexplained changes in therapeutic response, particularly during GLP-1RA initiation and dose escalation, and consider staggering oral medications when feasible.

Additionally, the common adverse effects of GLP-1RAs, such as nausea, constipation, and abdominal pain, frequently masquerade as an IBD flare. This symptom overlap easily confuses subjective scoring tools like the Crohn’s Disease Activity Index (CDAI) (109). To disentangle drug adverse effects from inflammatory activity, clinicians must employ a concrete diagnostic approach. Fecal calprotectin should be checked promptly when new gastrointestinal symptoms emerge. If symptoms are severe, endoscopic evaluation is warranted before escalating IBD therapies. Rather than stopping the GLP-1RA abruptly, clinicians should consider a temporary dose reduction while awaiting objective laboratory results.

## Ongoing clinical trials and future directions

To definitively answer whether GLP modulation offers true disease-modifying benefits, the field must rely on ongoing prospective trials (93, 103, 110). The COMMIT-CD program (NCT06937099) is a central piece of this translational narrative. This phase 3b trial evaluates mirikizumab combined with tirzepatide versus mirikizumab with a placebo in adult participants with moderately to severely active CD and comorbid obesity (111). The design of this anchor trial matters immensely because it compares the combined mechanistic approach directly against a standard biologic therapy. Endpoints focused on endoscopic healing will be required to demonstrate an additive benefit beyond weight loss. Mechanistic sampling of intestinal tissue during this trial would best validate the “reparative” hypothesis if analyses include tight junction protein expression (e.g., claudins, occludin, zonula occludens-1), histologic healing assessments using validated indices (e.g., Geboes score, Robarts Histopathology Index), and intestinal permeability assays such as the lactulose-mannitol ratio. Additionally, quantification of paracrine mediators such as IGF-1 and KGF in mucosal biopsies could directly link GLP pathway activation to the epithelial restitution programs identified in preclinical models.

Similar trials are assessing GLP-1RAs in overweight patients with IBD (110), in UC (NCT06937086), and exploring dual GLP-1/GIP agonists like Zepbound as adjunctive therapies (NCT06774079) (112). The role of tirzepatide in promoting intestinal healing via blood and stool samples is also being explored (113). Furthermore, phase 3 trials for GLP-2 analogs, such as glepaglutide and apraglutide, are ongoing for short bowel syndrome (114) and intestinal healing (115, 116). However, developers of GLP-2 therapies for chronic IBD will need to address prescriber concerns regarding long-term trophic effects and the necessity for mucosal dysplasia surveillance. Future GLP-2 trials in IBD should stratify patients by disease phenotype (mucosal UC, inflammatory CD, stricturing CD) and incorporate serial cross-sectional imaging to monitor for stricture progression, given the mechanistic link between GLP-2R signaling through subepithelial myofibroblasts and intestinal fibrogenesis. Finally, the development of dual GLP-1/GLP-2 receptor agonists, such as dapiglutide, has shown promise in reducing intestinal transit time and increasing villus height in murine models, representing an exciting future strategy (117).

## Conclusion

The exploration of GLP-1 and GLP-2 pathways has provided an emerging hypothesis and therapeutic rationale for intestinal repair in IBD. Preclinical data strongly map these peptides to enhanced barrier function, mucosal proliferation, and local immunomodulation. While current human observational data provide reassuring safety profiles and highlight metabolic advantages, true evidence for intrinsic disease modification remains limited and heavily confounded by systemic weight loss. The future of this therapeutic class in IBD management depends on rigorous prospective trials, such as the COMMIT-CD program, to validate endoscopic healing endpoints and establish standardized protocols for monitoring clinical friction points. By navigating these translational hurdles, GLP modulation may be successfully integrated into future disease-modifying treatment paradigms.
